# Qiyusanlong Formula Induces Autophagy in Non-Small-Cell Lung Cancer Cells and Xenografts through the mTOR Signaling Pathway

**DOI:** 10.1155/2021/5575453

**Published:** 2021-05-22

**Authors:** Yating Gao, Xinheng Wang, Qinjun Yang, Xiaole Wang, Xingxing Zhang, Jiabing Tong, Cheng Yang, Di Wu, Zegeng Li

**Affiliations:** ^1^Graduate School, Anhui University of Chinese Medicine, Hefei, Anhui, China; ^2^Institute of Traditional Chinese Medicine of Respiratory Disease Prevention, Anhui University of Chinese Medicine, Hefei, Anhui, China; ^3^The First Affiliated Hospital of Anhui University of Chinese Medicine, Hefei, Anhui, China

## Abstract

**Objective:**

Qiyusanlong (QYSL) formula has been used in the clinic for more than 20 years and has been proved to have pronounced efficacy in the treatment of non-small-cell lung cancer (NSCLC). This work aims to evaluate the molecular mechanism of QYSL formula action on NSCLC, specifically in relation to autophagy induction.

**Methods:**

In vitro, CCK-8 was used to detect the effect of QYSL serum on cell viability in A549 cells. In vivo, A549 cells were implanted subcutaneously in nude mice to establish a xenograft model. TUNEL staining was used to measure cell apoptosis and TEM to observe the autophagy-related morphological changes in vitro and in vivo. Western blotting, RT-qPCR, and immunofluorescence were used to measure autophagy-related proteins. In addition, rapamycin (an inhibitor of mTOR and inducer of autophagy) and MHY1485 (an activator of mTOR and inhibitor of autophagy) were used to determine whether QYSL-induced autophagy was regulated by the mTOR pathway.

**Results:**

QYSL serum inhibited the cell viability of A549 cells in a concentration‐dependent manner. In vivo, the QYSL formula inhibited xenograft growth. The QYSL formula promoted apoptosis in A549 cells and induced autophagosome formation in vitro and in vivo. In addition, the QYSL formula downregulated the expression of mTOR and p62, while it upregulated the expression of ATG-7 and Beclin-1 and increased the LC3-II/LC3-I ratio. QYSL serum inhibited p-mTOR in a similar manner to rapamycin while reducing the activating effects of MHY1485 on p-mTOR.

**Conclusion:**

The QYSL formula has anti-lung cancer effects and promotes autophagy through the mTOR signaling pathway.

## 1. Introduction

Lung cancer is the main cause of cancer morbidity and mortality in the world [1] and is also the main cause of cancer death in China [2]. Non-small-cell lung cancer (NSCLC) accounts for 85% of all lung cancers [3]. Although treatments have improved in recent years, the prognosis of NSCLC patients is still poor [4, 5]. Therefore, it is essential to find new antitumor drugs with high efficacy and low toxicity.

Traditional Chinese medicine (TCM) enjoys a long history in the prevention and treatment of disease [6], including lung cancer [7–10]. Qiyusanlong (QYSL) formula, created by Professor Han Mingxiang, has been applied in the clinic for more than 20 years and has been proved to have pronounced efficacy in lung cancer [11]. The formula consists of ten Chinese medicinal herbs: Astragalus, *Solanum nigrum*, Gecko, Earthwormg, *Euphorbia helioscopia*, *Hedyotis diffusa* Willd, *Curcuma zedoaria*, *Coix lacryma*, *Polygonatum*, and *Fritillaria cirrhosa* [12]. The untargeted metabonomics method based on UPLC-QTOF/MS has been used to screen out 21 biomarkers that may contribute to the treatment of lung cancer with the QYSL formula; the biomarkers were found to be mainly involved in sphingolipid and glycerophospholipid metabolism and fatty acid degradation [13]. Previous animal experiments have shown that the QYSL formula has tumor-suppressive properties and can decrease the expression of mTOR, a key protein in the PI3K/Akt/mTOR signaling pathway that is involved in autophagy, suggesting that QYSL's anticancer effect may be related to the promotion of autophagy in lung cancer cells [14].

Autophagy is an intracellular catabolic process that transfers cytosol and specific cellular contents, including dysfunctional organelles and pathogens, to the lysosome for degradation [15]. Autophagy is a mechanism for the recycling and reuse of material and can lead to apoptotic cell death; malfunctioning of these processes can participate in the development and progression of lung cancer [16], suggesting new methods for treating NSCLC. To date, several signaling pathways regulating autophagy have been identified, including mTOR, PI3K/AKT, and ROS signaling pathways, among which the mTOR pathway plays a significant role in cancer-related autophagy [17]. Inhibition of mTOR-related autophagy has been shown to have potential for cancer treatment and prevention [18].

In this study, we aimed to elucidate the molecular mechanism of action of the QYSL formula in treating NSCLC to provide new insights into the role of the QYSL formula in the induction of autophagy.

## 2. Materials and Methods

### 2.1. Drugs and Reagents

#### 2.1.1. Drugs

All the Chinese herbs in the QYSL formula, including 30 g Astragalus, 20 g *Solanum nigrum*, 6 g Gecko, 6 g Earthwormg, 6 g *Euphorbia helioscopia*, 20 g *Hedyotis diffusa* Willd, 10 g *Curcuma zedoaria*, 20 g *Coix lacryma*, 10 g *Polygonatum*, and 6 g *Fritillaria cirrhosa*, were purchased from the Pharmacy of Traditional Medicine in the First Affiliated Hospital of Anhui University of Chinese Medicine. The water decoction of the QYSL formula was condensed to a concentration of 4.024 g/mL and 1.396 g/mL, filtered through a 0.22 *μ*m filter, and stored at 4°C. This decoction was used in the effect of QYSL serum and tumor xenograft experiments, respectively.

#### 2.1.2. Reagents

Fetal bovine serum (FBS) (10270-106) was purchased from Gibco (NY, USA); high-glucose DMEM (SH30022.01B) from HyClone (USA); the CCK-8 assay kit from Dojindo (Japan); the TUNEL assay kit (11684795910) from Roche (Switzerland); 0.25% trypsin-EDTA (C0201), penicillin-streptomycin liquid (C0222), RIPA lysis solution (P0013B), PMSF (ST506), DAPI staining solution (C1006), and the BCA Protein Assay kit (P0012) from Beyotime Biotechnology (Shanghai, China); the SDS-PAGE Preparation kit (C831100-0200) and EZ-10 Total RNA Mini-Preps Kit (B618583) from Shenggong Biotech Inc. (Shanghai, China); SDS (S8010), glycine (G8200), and Tris (T8060) from Beijing Suo Laibao Technology Co., Ltd. (Beijing, China); PageRuler prestained marker (26616) from Thermo Fisher Scientific (MA, USA); anti-Beclin-1 (ab20761) and anti-ATG-5 (ab108327) antibodies from Abcam (Cambridge, UK); anti-p62 (88588S), anti-LC3 (3868S), anti-mTOR (2983T), and anti-p-mTOR (5536T) antibodies from Cell Signaling Technology (Danvers, MA, USA); ABScript II RT Master Mix for qPCR with gDNA Remover (RK20403) from ABclonal (Wuhan, China); TB Green™ Premix Ex Taq™ II (Tli RNase H Plus) (RR820A) from TaKaRa (Dalian, China); rapamycin (HY-10219) from MedChemExpress (MCE, NJ, USA); and MHY1485 (S7811) from Selleck (Houston, TX, USA).

### 2.2. Cells and Cell Culture

A549 cells were purchased from Procell Life Technology Co., Ltd. (Wuhan, China). The cells were cultured in a complete medium consisting of high-glucose DMEM, 10% FBS, and 1% penicillin and streptomycin in an incubator with 5% CO_2_ at 37°C. Cells were subcultured by removal from the flask surfaces with 0.25% EDTA/trypsin solution when 70%–80% confluent, terminating the reaction with the 10% FBS DMEM complete medium. The cells were diluted with medium, blown gently, and transferred into the new cell culture flask for subculture.

### 2.3. Preparation of Medicated Sera of QYSL

Forty male Sprague Dawley (SD) rats (weight 200–250 g) were obtained from the Anhui Medical University (Anhui, China). Animals were housed at the Anhui University of Chinese Medicine (AHUCM). Animals were randomly divided into a QYSL group (*n* = 20) and a blank group (*n* = 20). The animals of the QYSL group received intragastric doses of 1.396 g/mL QYSL twice a day for five days. Animals in the control group received doses of saline administered in the same manner on the same schedule. One hour after the last gavage, all animals were anesthetized by intraperitoneal injection of 3.5% pentobarbital sodium (10 ml/kg). The blood was collected by the abdominal aorta method in aseptic conditions. The serum was extracted by centrifugation at 1000 rpm for 5 min after standing for 1 h resting, was complement-inactivated at 56°C for 30 min in a water bath kettle, filtered through a 0.22 *μ*m membrane, and marked as blank serum or QYSL serum. The serum was stored in a −80°C freezer before use in cell culture experiments.

### 2.4. CCK-8 Assay

A549 cell suspensions were seeded in 96-well microplates at 100 *μ*L/well with a density of 5 × 10^4^/mL. When the cells were adherent, QYSL serum (at concentrations of 10%, 15%, 20%, 25%, and 30%) was added to the culture medium for 12 h, 24 h, or 48 h. After aspiration of the culture medium, 10 *μ*L of CCK-8 solution was added to each well; and after incubation for 1 h, the absorbance values were measured with a microplate reader at 450 nm. The experiment was repeated at least three times. The cell viability and IC_50_ values were then calculated.

### 2.5. Cell Treatments

A549 cells were treated with blank serum and QYSL serum (at concentrations of 20%) for 24 h. For inhibitor and activator experiments, A549 cells were incubated with the mTOR inhibitor rapamycin (RAPA) (100 nM) or the mTOR activator MHY1485 (2 *μ*M) for 1 h followed by treatment with QYSL serum for 24 h.

### 2.6. TUNEL Assay

Cells were cultured overnight on circular glass slides in 6-well plates. After being exposed to blank serum and QYSL serum for 24 h, cell slices were fixed with 4% paraformaldehyde for 20 min. After washing with PBS, TUNEL staining was performed according to the protocol. The reagents 1 (TdT) and 2 (dUTP) were mixed in a 1 : 9 ratio and then incubated with the slices at 37°C for 2 h away from light. The slices were then stained with DAPI solution for 10 min away from light at room temperature and were observed and photographed under fluorescence microscopy. The cells showing green fluorescence were classified as apoptotic cells. The ratio of apoptotic cells to total cells counted was quantified using Image J software.

### 2.7. Transmission Electron Microscopy

A549 cells were exposed to either blank serum or QYSL serum for 24 h and then collected by gentle scraping after fixation with electron microscope fixing solution for 2 h at 4°C. The cell pellet was collected after centrifugation at 2500 rpm for 2 min. Fresh tumor tissues from the mouse xenograft models (see Section 2.11 below) in each group were fixed immediately in electron microscope fixing solution for 4 h at 4°C. The samples were then fixed in 1% aqueous osmium at room temperature for 2 h and dehydrated using graded ethanol solutions (30, 50, 70, 80, 90, and 100%). Samples were then embedded in araldite. Ultrathin sections of 60–80 nm were cut with an ultramicrotome and were stained with 2% uranyl acetate and lead citrate for 15 min, then dried overnight. The images were observed and photographed under a transmission electron microscope (TEM).

### 2.8. Western Blot Analysis

Total proteins were extracted from cells and tumor tissue samples using RIPA lysis buffer according to the protocol. Protein quantification was performed by the BCA assay. The separation adhesive and concentration adhesive were prepared and the proteins were loaded, then transferred onto PVDF membranes. After transfer, the membranes were blocked for 2 h at room temperature with 5% skim milk. The membranes were then incubated with primary antibodies overnight at 4°C. The dilution ratio of primary antibody was as follows: ATG-7 (1 : 1000), ATG-5 (1 : 1000), p62 (1 : 1000), ATG-13 (1 : 1000), Beclin-1 (1 : 1000), LC3 (1 : 1000), mTOR (1 : 1000), p-mTOR (1 : 1000), or *β*-actin (1 : 1000). Next, the membranes were soaked with Tris-buffered saline containing 0.1% Tween-20 (TBST) and incubated with horseradish peroxidase-conjugated secondary antibodies for 1 h at room temperature. Immunoreactive bands were determined by the ECL kit and an imaging system. Image J software was used for quantitative analysis.

### 2.9. Quantitative Reverse-Transcription Polymerase Chain Reaction (qRT-PCR)

Total RNA from A549 cells was extracted using the EZ‐10 Total RNA Mini-Preps Kit according to the protocol. The concentration and purity of the total RNA were detected by an ultraviolet spectrophotometer. Then, the RNA was reversely transcribed into cDNA using the ABScript II RT Master Mix for qPCR with gDNA remover. The primers were synthesized by Hefei Deir Spectrum Biotechnology Co., Ltd (Anhui, China). The primer sequences are listed in Table 1, with *β*-actin used as the internal reference. Subsequently, qPCR was conducted using TB GreenTM Premix Ex TaqTM II, and relative gene expression was determined on an ABI 7300 machine. The relative expressions of mRNA were calculated by the 2^−△△Ct^ method.

### 2.10. Immunofluorescence Assay

Cell slices were prepared as described above (Section 2.6). After exposure to blank serum or QYSL serum for 24 h, the slices were fixed with 4% paraformaldehyde for 25 min and then blocked in 3% BSA. The slices were exposed to the appropriate primary antibodies overnight at 4°C followed by incubation with fluorescent-labeled secondary antibodies for 1 h away from light at room temperature. After washing with PBS, the slices were labeled with DAPI solution and incubated for 10 min away from light at room temperature. Finally, the slices were evaluated and photographed using fluorescence microscopy.

### 2.11. Tumor Xenograft Experiments

Forty male nude BALB/c mice aged 4 weeks weighing 14–16 g were purchased from Beijing Vital River Laboratory Animal Technology Co., Ltd. (Beijing, China) and raised in an SPF-level barrier environment at the Animal Center within the Anhui Medical University. Mice were allowed to adapt for one week. A549 cells were suspended to a concentration of 1 × 10^7^ cells/mL, and approximately 2 × 10^6^ cells per 200 *μ*L DMEM were injected subcutaneously into the flanks of the nude mice. When the average tumor volume reached 50–100 mm^3^, the mice were randomly divided into four groups (*n* = 10): (a) model group in which mice received 0.1 mL/10 g physiological saline via intragastric administration daily for two weeks; (b) QYSL group in which mice received 80.48 g/kg QYSL via intragastric administration daily for two weeks, as described in a preliminary study of the effective dose [12]; (c) RAPA group in which mice received 2 mg/kg RAPA [19] via intraperitoneal injection daily for two weeks; and (d) combined group in which mice received 80.48 g/kg QYSL [12] via intragastric administration and 2 mg/kg RAPA [19] via intraperitoneal injection daily for two weeks. The tumor volumes were measured using an electronic digital caliper every two days. At the end of the treatment period, the mice were euthanized, and the tumor tissues were isolated and measured with a precision electronic balance.

### 2.12. Statistical Analysis

SPSS (Version 23.0, IBM Corp., Armonk, NY, USA) was used for statistical analysis. The graphs were drawn using Prism 8. Data were expressed as mean ± SD (‾*X* ± *s*). Comparisons between two groups with homogeneous variance were analyzed using unpaired *t*-tests for independent samples. Comparisons between multiple groups were analyzed using one-way ANOVA. *P*-values less than 0.05 were considered to be statistically significant, and *P*-values less than 0.01, highly significant.

## 3. Results

### 3.1. QYSL Formula Inhibited the Cell Viability and Promoted Apoptosis in A549 Cells

**Figure 1:**
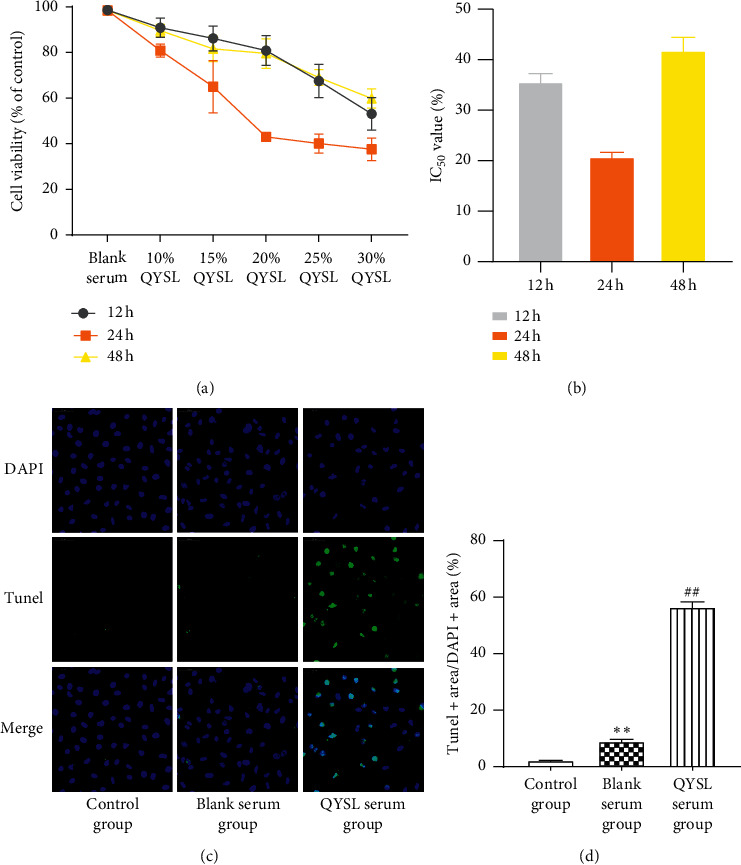
QYSL formula inhibited cell viability and promoted apoptosis in A549 cells. (a) The cell viability in A549 cells treated with QYSL serum at concentrations of 10%, 15%, 20%, 25%, and 30% for 12 h, 24 h, and 48 h was detected by the CCK-8 assay. (b) IC_50_ values of QYSL serum for different time points: 12 h (column 1), 24 h (column 2), and 48 h (column 3). (c) Representative images of TUNEL staining in A549 cells. (d) Quantitative analysis of TUNEL staining in A549 cells. Apoptosis in A549 cells exposed to culture medium (column 1), medium containing 20% blank serum (column 2), and medium containing 20% QYSL serum (column 3) was assessed by the TUNEL assay. ^*∗∗*^*P* < 0.01 vs. the control group. ^##^*P* < 0.01 vs. the blank serum group.

First, we examined whether exposure to QYSL serum affected the viability of A549 cells. After the cells were treated with QYSL serum at concentrations of 10%, 15%, 20%, 25%, and 30% for 12 h, 24 h, and 48 h, the CCK-8 assay was performed. Cells treated with QYSL serum showed a dose-dependent reduction in viability compared with those treated with blank serum (Figure 1(a)). Then, we calculated the IC_50_ value of the QYSL serum, which was found to be 20% for 24-h incubation, and this value was used for the following experiments (Figure 1(b)).

Cell apoptosis in A549 cells treated with QYSL serum was detected by the TUNEL assay. TUNEL staining indicated that the number of TUNEL-positive A549 cells in the QYSL group was significantly increased, compared with control and blank serum groups (Figure 1(c) and 1(d)), suggesting that the QYSL formula promoted apoptosis in A549 cells.

### 3.2. QYSL Formula Induced Autophagy in A549 Cells

**Figure 2:**
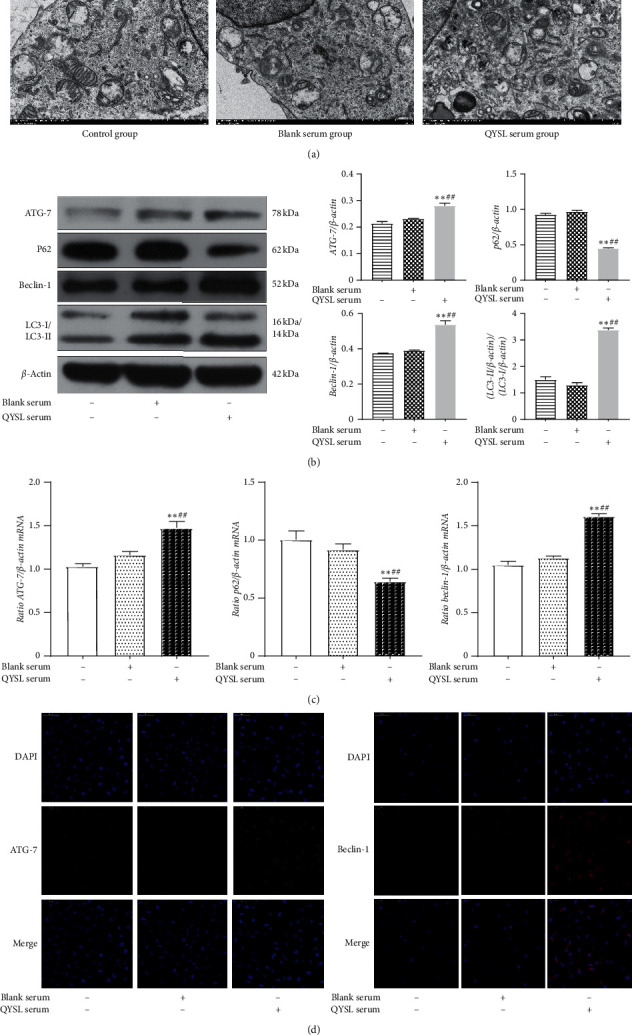
QYSL formula induced autophagy in A549 cells. A549 cells were exposed to the medium containing 10% FBS, 20% blank serum, or 20% QYSL serum for 24 h (a) QYSL formula induced morphological changes associated with autophagy in A549 cells, shown by TEM. (EM × 20000, 1.0 *μ*m). (b) Representative images and quantitative analysis of western blot showing protein levels of ATG-7, p62, Beclin-1, and LC3II/I in A549 cells. (c) The mRNA expression of ATG-7, p62, and Beclin-1 in A549 cells. (d) Representative images of IF showing the expression of ATG-7 and Beclin-1 in A549 cells. ^*∗∗*^*P* < 0.01 vs. the control group. ^##^*P* < 0.01 vs. the blank serum group.

Next, we investigated the presence of autophagic vacuoles by TEM, the standard technique for autophagy detection. When A549 cells were treated with QYSL serum, the number of cytoplasmic vacuoles with double-layered membrane structures increased. Many of these vacuoles were large and contained cytoplasmic organelles, which were not seen in either the control or the blank serum groups (Figure 2(a)). These morphological changes indicated that the QYSL formula might induce autophagosome formation in A549 cells.

To explore the exact effect of QYSL serum on inducing autophagy in A549 cells, autophagy-related molecules, including ATG-7, Beclin-1, p62, and LC3-II/LC3-I, were examined by western blotting, RT-qPCR, and immunofluorescence. These analyses showed that the expression of ATG-7 and Beclin-1 were significantly upregulated in the QYSL group, compared with the control and blank serum groups (Figure 2(b)–2(d)). Western blotting and RT-qPCR studies showed that the expression of p62 was significantly downregulated in the QYSL group (Figure 2(b) and 2(d)). Western blotting showed that the LC3-II/LC3-I ratio in the QYSL group was significantly increased (Figure 2(b)). The results suggested that the QYSL formula induced autophagy in A549 cells.

### 3.3. QYSL Formula Induced Autophagy in A549 Cells by the mTOR Signaling Pathway

**Figure 3:**
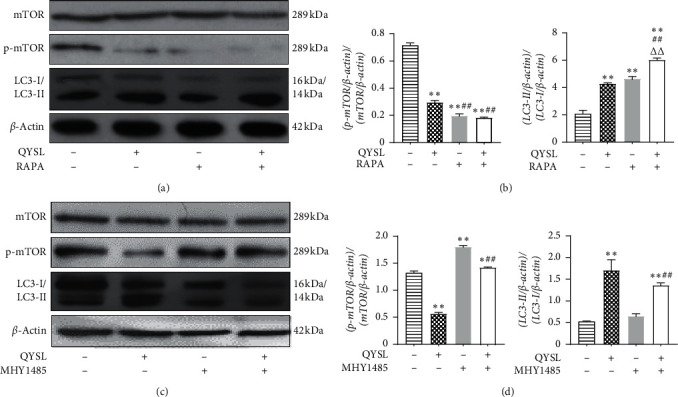
QYSL formula induced autophagy in A549 cells by inhibiting the mTOR pathway. (a) Representative images of western blot bands showing protein levels in A549 cells treated with QYSL serum in the absence or presence of RAPA for 24 h. (b) The densitometry values of western blot bands in A549 cells treated with QYSL serum in the absence or presence of RAPA were normalized to *β*-actin and represented as relative intensity. (c) Representative images of western blot showing protein levels in A549 cells treated with QYSL serum in the absence or presence of MHY1485 for 24 h. (d) The densitometry values of western blot bands in A549 cells that were treated with QYSL serum in the presence or absence of MHY1485 were normalized to *β*-actin and represented as relative intensity. ^*∗*^*P* < 0.05 and ^*∗∗*^*P* < 0.01 vs. the control group. ^##^*P* < 0.01 vs. the QYSL group. ^ΔΔ^*P* < 0.01 vs. the RAPA group.

Previous studies have shown that the QYSL formula can reduce the expression level of mTOR [14]. To determine whether the QYSL formula induced autophagy through the mTOR pathway, we used RAPA (an inhibitor of mTOR and inducer of autophagy) and MHY1485 (an activator of mTOR and inhibitor of autophagy). Similar to RAPA, QYSL serum inhibited p-mTOR and increased the expression of LC3-II/LC3-I compared with the control group (Figure 3(a) and 3(b), respectively). Meanwhile, QYSL serum lowered the activation of MHY1485 on p-mTOR in A549 cells and increased the expression of LC3-II/LC3-I, compared with the control and MHY1485 groups (Figure 3(c) and 3(d), respectively).

Taken together, these results indicated that the QYSL formula induced autophagy in A549 cells by inhibiting the mTOR pathway.

### 3.4. QYSL Formula Inhibited the Growth of NSCLC In Vivo

**Figure 4:**
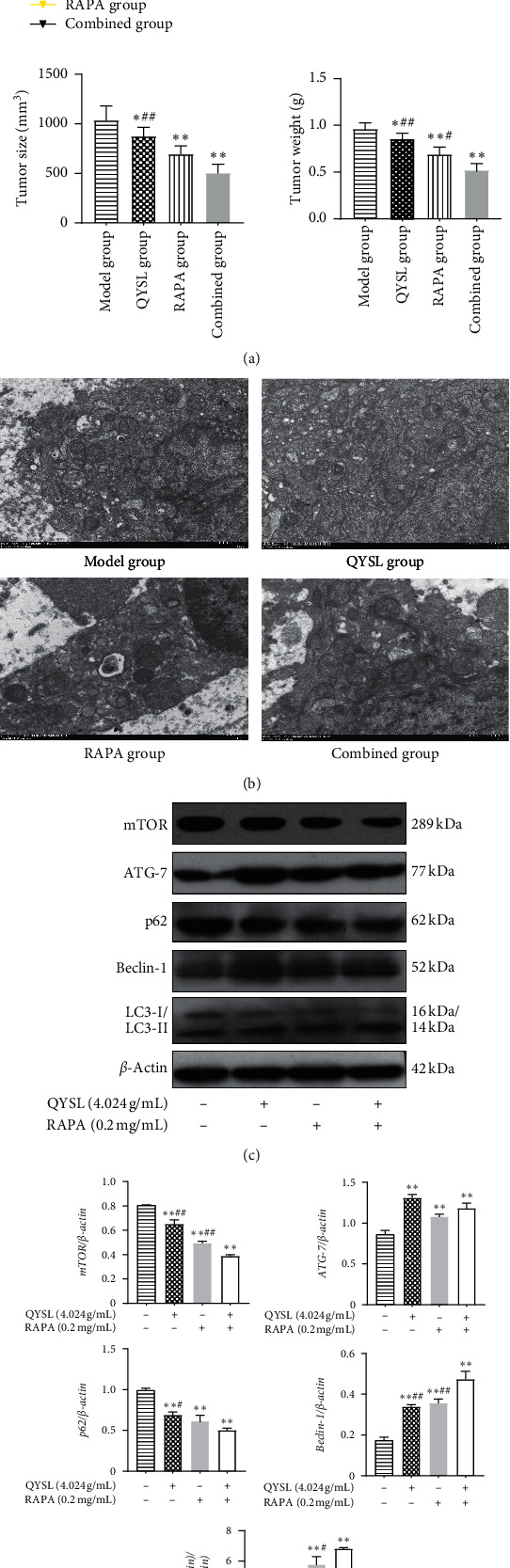
QYSL formula inhibited tumor growth of NSCLC in vivo. (a) QYSL formula inhibited the tumor growth of xenografts in nude mice. The tumor growth curve, representative tumor tissues, the final average tumor volumes, and tumor weights in each group are shown. (b) Morphological changes associated with autophagy induced by the QYSL formula in tumor tissues. The nude mice were treated with saline (model group), QYSL formula (QYSL group) or RAPA (RAPA group), or QYSL formula and RAPA (combined group). The morphological changes of tumor tissues in each group were detected by TEM. (EM × 20000, 1.0 um). (c) Representative images of western blot showing protein expression of mTOR, ATG-7, p62, Beclin-1, and LC3-II/LC3-I in tumor tissues in vivo. (d) The densitometry values of the bands were normalized to *β*-actin and represented as relative intensity. ^*∗*^*P* < 0.05 and ^*∗∗*^*P* < 0.01 vs. the model group. ^#^*P* < 0.05 and ^##^*P* < 0.01 vs. the combined group.

Based on the results in vitro, we established a xenograft model of NSCLC in BALB/c-nude mice using A549 cells to verify the tumor-suppressive role of the QYSL formula in vivo (Figure 4(a)). The tumor volume was measured every two days, and it was observed that, while the tumor volumes in each group increased continuously, the mean volumes in the QYSL group, RAPA group, and combined group were significantly reduced compared with the model group (*P* < 0.05). The tumor tissues were separated and photographed after sacrifice and then weighed, which showed that both the tumor volume and weight were significantly decreased in the QYSL group (*P* < 0.05), RAPA group (*P* < 0.01), and combined group (*P* < 0.01), compared with the model group. The results indicated that the QYSL formula could suppress NSCLC tumor growth.

The morphological changes of the tumor tissue in each group were observed using TEM. As shown in Figure 4(b), the cells from the model group exhibited few autophagic vesicles and showed intact nuclear and organelle structures. The numbers of intracellular autophagic vacuoles visible as double-membrane vesicles were increased in the QYSL and RAPA groups compared with the model group, and it were significantly increased in the combined group.

To further elucidate the mechanism by which the QYSL formula inhibited tumor growth in vivo, autophagy-related proteins were detected by western blotting analysis. The protein expression of mTOR and p62 were markedly downregulated, and the protein expression of ATG-7, Beclin-1, and LC3-II/LC3-I were markedly upregulated in the tumor tissues in the QYSL and RAPA groups compared with the model group (Figure 4(c) and 4(d)). These results suggested that tumor growth inhibition is associated with QYSL-induced autophagy in vivo.

## 4. Discussion

The QYSL formula has noticeable effects on the prevention and treatment of NSCLC [12, 13]. Our previous studies showed that the anticancer properties of the QYSL formula may be associated with autophagy although the specific mechanism was not yet clear. Therefore, we examined the antitumor effects of the QYSL formula and its mechanisms in vitro and in vivo in this study.

Autophagy is an intracellular catabolic process that involves cell metabolism and the recycling of damaged and dysfunctional organelles and proteins [20–22]. The role of autophagy in lung cancer remains controversial. On the one hand, autophagy has been found to be a cell survival mechanism in which cells can survive under metabolic stress by degrading and clearing damaged organelles and cytoplasmic proteins through the lysosomes [23]. However, autophagy dysfunction can inhibit the activity of cancer cells or play an indirect antitumor role by inducing apoptosis [24]. Therefore, the regulation of autophagy is an effective interventional strategy for the treatment of lung cancer [22, 25]. The TUNEL staining can be used to assess apoptosis [26, 27]. In our study, the TUNEL staining showed that the QYSL formula induced cell apoptosis in A549 cells.

To verify the effects of the QYSL formula on autophagy of A549 cells, autophagic structures and autophagy-related markers were measured using TEM, together with RT-qPCR and western blotting. The presence of the characteristic double-membrane autophagic vacuoles or autophagosomes in TEM is the standard for detecting autophagy [28]. In particular, the morphological changes associated with autophagosome formation were observed under TEM, which suggested that the QYSL formula induced autophagosome formation in A549 cells.

As a tumor suppressor gene, Beclin-1 is a crucial modifier of autophagy and is involved in the activation of autophagy and the formation of autophagosomes [29–31]. LC3 and p62, the key proteins in autophagosome formation, are commonly used as autophagic markers [32]; p62 can be recruited to the autophagosomal membrane through binds interaction with LC3 [33]. In the absence of autophagy, LC3B is processed to the soluble LC3B-I, and once autophagy has occurred, LC3B-I is activated by ATG-7 and binds to phosphatidylethanolamine, thereby transforming into LC3B-II [34]. The amount of LC3B-II present in the cell is, therefore, specifically related to the degree of autophagy [35]. In our study, the expression of p62 was markedly downregulated, while the expression of Beclin-1, ATG-7, and LC3-II/LC3-I was simultaneously upregulated following treatment with the QYSL formula. The morphological characteristics of autophagosome formation observed through TEM and the changes of autophagy-related genes and proteins provide strong evidence of autophagy induced by the QYSL formula in A549 cells.

Autophagy is regulated by a number of upstream signaling pathways, including the mTOR pathway that is closely associated with autophagy induction [36–38]. There is growing evidence that the mTOR signaling pathway plays an important role in the occurrence and development of many malignant cancers [39–44], including lung, breast, stomach, and liver cancer. mTOR is a key regulatory factor in the initiation stages of autophagy and functions as a negative regulatory molecule of autophagy, so that its activation inhibits autophagy [45]. mTOR divided into two distinct multiprotein complexes, namely mTORC1 and mTORC2, with autophagy being mainly controlled by mTORC1 [31]. Research has shown that inhibition of mTORC1 increases autophagy, while the activation of mTORC1 reduces autophagy [46]. The negative regulation of autophagy by mTORC1 occurs mainly through ATG-13 [47]. Inhibition of mTOR is, therefore, an important therapeutic target for cancer treatment [48, 49].

To further elucidate the mechanism by which the QYSL formula induced autophagy in A549 cells, we used the autophagy inhibitor MHY1485 and the autophagy activator RAPA. Similar to RAPA, QYSL inhibited mTOR phosphorylation and increased the ratio of LC3II to LC3I. After activation of the mTOR pathway by MHY1485, the expression of p-mTOR was significantly increased, while the QYSL formula significantly suppressed both mTOR phosphorylation and the increase in the LC3II/LC3I ratio. The results indicated that the QYSL formula induced autophagy in A549 cells by the mTOR signaling pathway.

Based on the results in vitro, we further verified the tumor-suppressive effects of the QYSL formula in vivo by the construction of xenograft tumors in BALB/c nude mice with A549 cells. The mean tumor volumes and weights in the QYSL group were significantly lower than those in the model group. The morphological changes of autophagosome formation observed through TEM and the altered expression of autophagy-related molecules provide strong evidence that the QYSL formula induced autophagy in vivo.

In conclusion, both the in vitro and in vivo results demonstrated that the QYSL formula has antitumor effects, and its mechanism is involved in autophagy via the mTOR signaling pathway. These findings suggest the potential of the QYSL formula in the treatment of NSCLC.

## Figures and Tables

**Table 1:** Related gene sequence primers.

Primer name	Sequences (5′ to 3′)	Length (bp)
Beta-Actin-F	CCTCACTGTCCACCTTCC	120
Beta-Actin-R	GGGTGTAAAACGCAGCTC	

ATG-7-F	TTTGCTATCCTGCCCTCTGTC	199
ATG-7-R	TTAAGCAAGGAAACCAGCACC	

ATG-5-F	GAAGCTGTTTCGTCCTGTGG	186
ATG-5-R	TCCGGGTAGCTCAGATGTTC	

ATG-13-F	GCTGCTGAAGTCCCTTCTTG	144
ATG-13-R	ACTGTCTGGAAGCCTTCTCC	

Beclin-1-F	AACCTCAGCCGAAGACTGAA	136
Beclin-1-R	CAGTGACGTTGAGCTGAGTG	

p62-F	GTCTGCGAGGGAAAGGG	126
p62-R	CCCGAAGTGTCCGTGTTT	

## Data Availability

All data used to support the findings of this study are included within the article.
